# Two successful deliveries following fertility-sparing treatment for synchronous endometrial and ovarian cancer: a case report and literature review

**DOI:** 10.1007/s13691-026-00867-1

**Published:** 2026-04-07

**Authors:** Mamiko Ohta, Kosuke Murakami, Kazuyoshi Kato, Noriomi Matsumura

**Affiliations:** 1 Department of Obstetrics and Gynecology, Kindai University Faculty of Medicine, 1-14-1 Miharadai, Minami-ku,, Sakai, Osaka 590-0197 Japan; 2 Department of Gynecology, Kitasato University School of Medicine, Sagamihara, Kanagawa Japan

**Keywords:** Endometrial cancer, Fertility-sparing treatment, Ovarian cancer, Synchronous cancer

## Abstract

Fertility-sparing treatment is a viable treatment option for selected patients with endometrial or ovarian cancer. However, its use in synchronous endometrial and ovarian cancer (SEOC) remains rare, and standardized treatment strategies have yet to be established. We report the case of a 26-year-old woman diagnosed with SEOC, characterized as grade 1, stage IA endometrioid carcinoma of both the endometrium and ovary. Genetic analysis ruled out hereditary cancer syndromes, including Lynch syndrome. The patient was treated with medroxyprogesterone acetate for 26 weeks, after which histological evaluation confirmed complete remission. She subsequently achieved two spontaneous pregnancies, both resulting in uncomplicated vaginal deliveries. To our knowledge, this is the fifth reported case of childbirth following fertility-sparing treatment for SEOC and the first involving two successful deliveries.

## Introduction

In recent years, the incidence of endometrial and ovarian cancers has increased in Japan [1]. Synchronous endometrial and ovarian cancer (SEOC) accounts for approximately 5% of endometrial cancers and 10% of ovarian cancers [2]. SEOC typically carries a favorable prognosis [3] and has traditionally been regarded as a distinct clinical entity rather than a manifestation of metastatic disease [4]. However, recent next-generation sequencing (NGS) studies have shown that most SEOCs are not independent primary tumors, but rather represent metastasis from endometrial cancer to the ovary [3, 5]—except in cases associated with Lynch syndrome [6].

Fertility-sparing treatment is an established option for well-selected cases of grade 1 endometrioid carcinoma confined to the endometrium, particularly in young women [7, 8]. Similarly, it is considered acceptable for stage IA mucinous and endometrioid ovarian carcinomas with low-grade histologic features [7, 9]. Reported pregnancy rates following fertility-sparing treatment range from 25 to 66% for endometrial cancer and 30 to 54% for ovarian cancer. Among patients actively attempting conception, the rates are even higher—93.3% for endometrial cancer and 89% for ovarian cancer [10]. Despite these favorable outcomes, reports of fertility-sparing treatment for SEOC—in which both tumors independently fulfill the criteria for conservative management—remain extremely limited. As a result, the feasibility and reproductive outcomes of such treatment in SEOC remain uncertain.

In this study, we report a rare case of SEOC in a young woman who achieved two successful spontaneous pregnancies and deliveries following fertility-sparing treatment. We also review the existing literature to provide further insight into the feasibility and outcomes of fertility preservation in SEOC. We believe this case offers valuable evidence to inform future guidelines on fertility-sparing management of SEOC.

### Case report

The patient was a 26-year-old nulligravid woman with a history of irregular menstrual cycles since menarche at age 12. She was 158 cm tall, weighed 49 kg, and had a body mass index (BMI) of 19.6. Her medical history included a left salpingectomy at age 13 due to torsion of a left tubal cyst. No significant family history of malignancy was identified among first- or second-degree relatives.

**Fig. 1:**
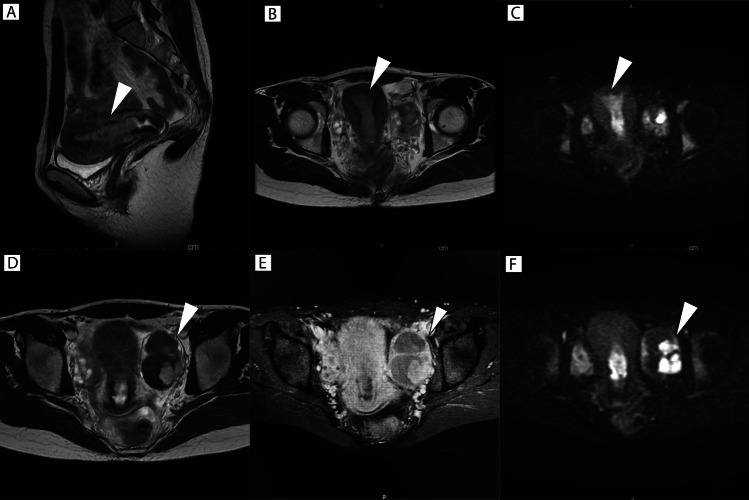
Pelvic magnetic resonance imaging findings at initial presentation. (**A**, **B**) Sagittal and axial T2-weighted images showing endometrial thickening (arrowheads). (**C**) Diffusion-weighted image showing restricted diffusion in the thickened endometrium (arrowhead), consistent with malignancy. (**D**–**F**) Axial T2-weighted, contrast-enhanced T1-weighted, and diffusion-weighted images, respectively, showing a left ovarian mass with cystic and solid components (arrowheads). The junctional zone is preserved, and no evidence of extrauterine or metastatic spread is observed

She initially presented to a local clinic with abnormal vaginal bleeding. Transvaginal ultrasound revealed endometrial thickening and a left ovarian tumor with solid components. She was referred to our institution for further evaluation. An endometrial biopsy revealed grade 1 endometrioid carcinoma. Pelvic contrast–enhanced magnetic resonance imaging (MRI) showed endometrial thickening measuring 3 cm (Figs. 1A and B) with restricted diffusion (Fig. 1C), while preservation of the junctional zone suggested that the tumor was confined to the endometrium. The left ovary was cystically enlarged to 5 cm and contained solid components (Figs. 1D–F). Chest–abdominal contrast–enhanced computed tomography (CT), as well as positron emission tomography–CT (PET–CT), revealed no lymph node involvement or distant metastasis. Complete endometrial curettage, laparoscopic left adnexal resection, and partial omentectomy were performed. Intraoperative findings revealed that the left fallopian tube had already been removed, and no abnormalities were observed in the right adnexa or on the uterine serosa. The ovarian tumor was resected intact via a 7-cm laparotomy without rupture.

**Fig. 2:**
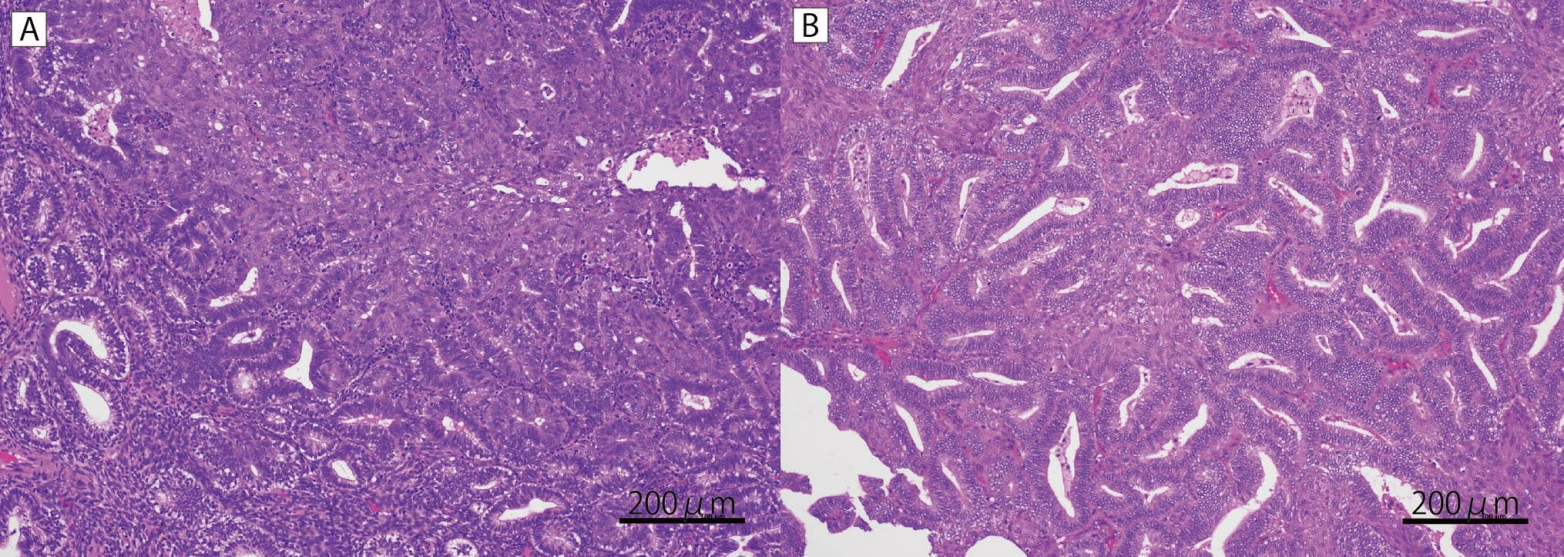
Histopathological findings of endometrial and ovarian tumors. Both tumors were diagnosed as grade 1 endometrioid carcinoma (**A**: endometrial lesion; **B**: ovarian lesion, H&E staining)

Postoperative pathological examination of the endometrial specimen confirmed grade 1 endometrioid carcinoma (Fig. 2A). The ovarian tumor was likewise diagnosed as grade 1 endometrioid carcinoma (Fig. 2B). No metastasis was found in the omentum, and peritoneal cytology was negative. The diagnostic criteria for distinguishing ovarian metastasis of endometrial cancer from SEOC include: (1) small ovarian size (< 5 cm), (2) bilateral ovarian involvement, (3) deep myometrial invasion, (4) lymphovascular space invasion, and (5) fallopian tube involvement [4]. In this case, only criterion (1) was met. Therefore, the diagnosis was SEOC, with stage IA endometrial cancer (The International Federation of Gynecology and Obstetrics (FIGO) 2008) and stage IA ovarian cancer (FIGO 2014). Although tumor-specific genetic or mutational analyses were not performed, the diagnosis of synchronous primary tumors was established based on widely accepted clinicopathological criteria, including those proposed by Ulbright and Roth and later modified by Scully. In the present case, both tumors were confined to their respective organs without lymphovascular space invasion, fallopian tube involvement, or peritoneal dissemination, supporting independent primary origins rather than metastatic disease. While recent studies have highlighted the potential role of molecular analyses in distinguishing synchronous primary tumors from metastatic disease, the clinicopathological findings in this case were considered sufficient to support the diagnosis of SEOC.

Given the patient’s young age at diagnosis, Lynch syndrome was considered. Immunohistochemistry for mismatch repair (MMR) proteins (MLH1, MSH2, MSH6, and PMS2) showed preserved expression of all markers. Additionally, germline NGS of peripheral blood detected no pathogenic variants. Multiplex ligation-dependent probe amplification (MLPA) analysis of *BRCA1*, *BRCA2*, *MLH1*, *MSH2*, and *EPCAM* also revealed no germline mutations.

At the time of surgery, only one case of fertility–sparing treatment for SEOC had been reported [11]. However, due to the patient’s strong desire to preserve fertility, definitive surgery was deferred. Instead, conservative management was initiated with medroxyprogesterone acetate (MPA) at 600 mg/day for 26 weeks. Complete remission was confirmed by total endometrial curettage performed under general anesthesia, demonstrating no residual carcinoma or atypical hyperplasia. Pelvic MRI and chest–abdominal CT confirmed the absence of residual disease or distant metastasis. MPA therapy was concluded as planned. Following treatment completion, endometrial biopsy was performed every 3–6 months, and thoracoabdominal CT was conducted annually as part of oncologic surveillance. The patient subsequently received six cycles of progesterone withdrawal therapy, each resulting in withdrawal bleeding. Treatment was discontinued in accordance with the patient’s desire to conceive. Two months later, she spontaneously conceived. The pregnancy progressed uneventfully, and she delivered a healthy female infant vaginally at 40 weeks and 3 days of gestation. The newborn weighed 2,892 g, with Apgar scores of 8 and 10 at one and five minutes, respectively.

Follow-up endometrial biopsies and CT revealed no evidence of recurrence. Two years and two months after the first delivery, the patient spontaneously conceived again. The second pregnancy also progressed without complications, resulting in a vaginal delivery at 39 weeks and 2 days. The second newborn, also female, weighed 3,000 g with Apgar scores of 8 and 10 at one and five minutes, respectively. No obstetric complications, including placenta accreta spectrum or excessive hemorrhage, were observed in either pregnancy, and placental histopathological examination was not performed.

The patient remains committed to fertility preservation and has not undergone hysterectomy. She has shown no signs of recurrence during the six years since completing MPA treatment.

## Discussion and literature review

A PubMed search was conducted using the terms “fertility-sparing therapy,” “ovarian carcinoma,” and “endometrial carcinoma.” After screening abstracts and applying filters as of March 1, 2025, five reports were identified that described pregnancy outcomes following fertility-sparing treatment for synchronous endometrial and ovarian cancer (SEOC) (Table 1). Case 1 involved laparoscopic resection of a left ovarian tumor, during which capsular rupture occurred. A second procedure with left adnexal resection and endometrial curettage was performed, followed by chemotherapy with carboplatin and cyclophosphamide. The patient then received hormonal therapy with megestrol acetate (MA) and leuprorelin acetate (LA), eventually achieving spontaneous pregnancy and vaginal delivery [11]. Case 2 involved a diagnosis of grade 2 endometrioid carcinoma of the endometrium. The patient underwent combined treatment with MA and LA, in addition to chemotherapy with paclitaxel and carboplatin. A right ovarian endometriotic cyst was also noted. Although intrauterine insemination failed, the patient achieved pregnancy via in vitro fertilization (IVF) using donor oocytes and delivered via cesarean Section [12]. In case 3, hormonal treatment with MA was initiated, but self-discontinued after two weeks. Eight months later, the patient presented with a spontaneous nine-week pregnancy and ultimately delivered vaginally at term [13]. Case 4 was derived from a case series comprising eight patients who underwent fertility-sparing management, including four patients with synchronous endometrial and ovarian cancer (SEOC), two with endometrial carcinoma and borderline ovarian tumors, and two with atypical endometrial hyperplasia and borderline ovarian tumors. The left ovarian tumor was successfully removed by cystectomy without capsular rupture. Following postoperative treatment with megestrol acetate (MA), pregnancy was achieved via in vitro fertilization and embryo transfer (IVF-ET). At 38 weeks of gestation, an elective cesarean section was performed, during which hysterectomy and bilateral adnexectomy were conducted. This represented the only reported pregnancy achieved after fertility-sparing treatment for SEOC within this case series [14].

**Table 1:** Pregnant cases following fertility-sparing management for SEOC

Case	Age	Stage and histology		MMR	Chemotherapy	Hormonal treatment	Pregnancy	Delivery	TAH	Follow-up after treatment completion	Recurrence	References
		uterus	ovary									
1	25	IA EM G1	IC1 EM	N/A	Car + Cyc	MA + LA	Spontaneous	Vaginal	No	33 months†	None	Atallah D, et al. [11]
2	30	IA EM G2	IA EM	N/A	TC	MA + LA	IVF with donor egg	C/S	No	30 months†	None	Suri V, et al. [12]
3	28	IA EM G1	IA EM	pMMR	No	MA*	Spontaneous	Vaginal	No	23 months†	None	Daley D, et al. [13]
4	28	IA EM G1	IA EM	pMMR	No	MA	Spontaneous	C/S	Yes	55.7 months	None	Gama Q, et al. [14]
5	26	IA EM G1	IA EM	pMMR	No	MPA	Spontaneous × 2	Vaginal × 2	No	68 months	None	This case

MMR, mismatch repair; TAH, total abdominal hysterectomy; EM, endometrioid carcinoma; G, grade; N/A, Not Available; Car, carboplatin; Cyc, cyclophosphamide; MA, megestrol acetate; LA, leuprolide acetate; TC, paclitaxel + carboplatin; IVF, in vitro fertilization; C/S, caesarean section; pMMR, MMR proficient; MPA, medroxyprogesterone acetatel.

* Hormonal therapy was self-discontinued by the patient after 2 weeks.

† Estimated from the clinical course described in the report

In Case 5, the present case, MPA, a hormonal agent approved in Japan, was used. MPA has been reported to have similar efficacy and safety to MA in fertility–sparing treatment [15]. As in Cases 3 and 4, mismatch repair (MMR) testing was performed to assess eligibility for conservative management. This was based on current evidence indicating that dMMR tumors tend to be resistant to progestin-based treatment [16, 17], that Lynch syndrome is more frequently associated with SEOC [18], and that patients with Lynch syndrome have a heightened risk of secondary ovarian malignancies [19]. Since MMR immunohistochemistry alone cannot definitively rule out Lynch syndrome [20, 21], germline genetic testing was also conducted, which identified no pathogenic variants. Within the framework of the ProMisE molecular classification, the present tumor could not be fully classified because *POLE* mutation analysis and p53 immunohistochemistry were not performed.

Fertility-sparing treatment for synchronous endometrial and ovarian cancer requires particularly careful patient selection and oncologic evaluation. Both tumors in the present case fulfilled the established criteria for conservative management, including grade 1 endometrioid histology and disease confined to the endometrium and ovary without extrauterine spread. Comprehensive assessment with imaging and pathological evaluation was performed before initiating hormonal therapy. In addition, close surveillance was implemented after treatment completion, including periodic endometrial biopsy and annual imaging. Assessment of MMR status is also important in patients with SEOC, given the higher prevalence of Lynch syndrome and the reported association between dMMR tumors and reduced response to progestin-based therapy.

Our case is unique in that it represents the first reported instance of two live births following fertility–sparing treatment for SEOC. While multiple live births after such treatment have been documented in patients with either endometrial or ovarian cancer individually [22–24], the feasibility of achieving multiple deliveries in SEOC has not been previously reported. This case suggests that, in carefully selected patients, fertility–sparing treatment may be a reasonable option even in SEOC. Given the potential risk of recurrence [22], definitive surgery—consisting of hysterectomy and adnexal resection—is planned once the patient no longer desires fertility preservation.

## Conclusion

Fertility-sparing treatment may be considered in cases of SEOC when both tumors independently meet the established criteria for conservative management. However, due to the rarity of such cases, supporting evidence remains limited. Additional case reports and long-term follow-up data are needed to better evaluate the safety, efficacy, and reproductive outcomes of fertility preservation in SEOC.

## References

[1] Nakai H, Higashi T, Kakuwa T, Matsumura N (2024) Trends in gynecologic cancer in Japan: incidence from 1980 to 2019 and mortality from 1981 to 2021. Int J Clin Oncol 29:363–371 38381162 10.1007/s10147-024-02473-8

[2] Chui MH, Ryan P, Radigan J et al (2014) The histomorphology of Lynch syndrome-associated ovarian carcinomas: toward a subtype-specific screening strategy. Am J Surg Pathol 38(9):1173–1181 25025451 10.1097/PAS.0000000000000298

[3] Anglesio MS, Wang YK, Maassen M et al (2016) Synchronous endometrial and ovarian carcinomas: evidence of clonality. J Natl Cancer Inst 108(6):djv428 26832771 10.1093/jnci/djv428

[4] Ulbright TM, Roth LM (1985) Metastatic and independent cancers of the endometrium and ovary: a clinicopathologic study of 34 cases. Hum Pathol 16:28–34 2982713 10.1016/s0046-8177(85)80210-0

[5] Reijnen C, Küsters-Vandevelde HVN, Ligtenberg MJL et al (2020) Molecular profiling identifies synchronous endometrial and ovarian cancers as metastatic endometrial cancer with favorable clinical outcome. Int J Cancer 147:478–489 32022266 10.1002/ijc.32907 PMC7317735

[6] Moukarzel LA, Da Cruz PA, Ferrando L et al (2021) Clonal relationship and directionality of progression of synchronous endometrial and ovarian carcinomas in patients with DNA mismatch repair-deficiency associated syndromes. Mod Pathol 34:994–1007 33328602 10.1038/s41379-020-00721-6 PMC8076061

[7] Nitecki R, Woodard T, Rauh-Hain JA (2020) Fertility-sparing treatment for early-stage cervical, ovarian, and endometrial malignancies. Obstet Gynecol 136(6):1157–1169 33156194 10.1097/AOG.0000000000004163 PMC7680432

[8] Rodolakis A, Scambia G, Planchamp F et al (2023) ESGO/ESHRE/ESGE Guidelines for the fertility-sparing treatment of patients with endometrial carcinoma. Hum Reprod Open. 2023(1):hoac057 36756380 10.1093/hropen/hoac057 PMC9900425

[9] Morice P, Scambia G, Abu-Rustum NR et al (2024) Fertility-sparing treatment and follow-up in patients with cervical cancer, ovarian cancer, and borderline ovarian tumours: guidelines from ESGO, ESHRE, and ESGE. Lancet Oncol 25(11):e602–e610 39216500 10.1016/S1470-2045(24)00262-6

[10] Contreras NA, Sabadell J, Verdaguer P, Julià C, Fernández-Montolí ME (2022) Fertility-sparing approaches in atypical endometrial hyperplasia and endometrial cancer patients: current evidence and future directions. Int J Mol Sci 23(5):2531 35269674 10.3390/ijms23052531 PMC8910633

[11] Atallah D, Safi J, El Kassis N, Rouzier R, Chahine G (2013) Simultaneous early ovarian and endometrial cancer treated conservatively with spontaneous pregnancy. J Ovarian Res 21(6):59 10.1186/1757-2215-6-59 PMC3765125 23965544

[12] Suri V, Bansal R, Aggarwal N et al (2023) Successful in vitro fertilization following conservative surgery for synchronous endometrioid tumor of ovary and uterus. J Ovarian Res 16(1):63 36991430 10.1186/s13048-023-01137-x PMC10053953

[13] Daley D, Padwick M, Mistry S, Malhotra V, Vikram RS, Stanciu P (2022) Case report: Spontaneous remission of synchronous endometrial and ovarian cancers following pregnancy. Front Oncol 29(12):1001677 10.3389/fonc.2022.1001677 PMC9745153 36523967

[14] Gama Q, Luo S, Wu P et al (2023) The pregnancy and oncology outcome of fertility-sparing management for synchronous primary neoplasm of endometrium and ovary. J Ovarian Res 16(1):235 38062462 10.1186/s13048-023-01316-w PMC10704711

[15] Park JY, Kim DY, Kim JH et al (2013) Long-term oncologic outcomes after fertility-sparing management using oral progestin for young women with endometrial cancer (KGOG 2002). Eur J Cancer 49(4):868–874 23072814 10.1016/j.ejca.2012.09.017

[16] Chung YS, Woo HY, Lee JY et al (2021) Mismatch repair status influences response to fertility-sparing treatment of endometrial cancer. Am J Obstet Gynecol 224(4):370.e1-370.e13 33039397 10.1016/j.ajog.2020.10.003

[17] Raffone A, Catena U, Travaglino A et al (2021) Mismatch repair-deficiency specifically predicts recurrence of atypical endometrial hyperplasia and early endometrial carcinoma after conservative treatment: a multi-center study. Gynecol Oncol 161(3):795–801 33812697 10.1016/j.ygyno.2021.03.029

[18] Rossi L, Le Frere-Belda MA, Laurent-Puig P et al (2017) Clinicopathologic characteristics of endometrial cancer in Lynch syndrome: a french multicenter study. Int J Gynecol Cancer 27:953–960 28525912 10.1097/IGC.0000000000000985

[19] Watson P, Vasen HFA, Mecklin JP et al (2008) The risk of extra-colonic, extra-endometrial cancer in the Lynch syndrome. Int J Cancer 123(2):444–449 18398828 10.1002/ijc.23508 PMC2627772

[20] Huth C, Kloor M, Voigt AY et al (2012) The molecular basis of EPCAM expression loss in Lynch syndrome-associated tumors. Mod Pathol 25(6):911–916 22388758 10.1038/modpathol.2012.30

[21] Clendenning M, Buchanan DD, Walsh MD et al (2011) Mutation deep within an intron of MSH2 causes Lynch syndrome. Fam Cancer 10(2):297–301 21360204 10.1007/s10689-011-9427-0 PMC4580736

[22] Fujiwara H, Jobo T, Takei Y et al (2012) Fertility-sparing treatment using medroxyprogesterone acetate for endometrial carcinoma. Oncol Lett 3(5):1002–1006 22783380 10.3892/ol.2012.602 PMC3389624

[23] Wang J, Fang Y, Chen T, Xin Z, Wu Y, Yang X (2024) A Case report of consecutive live birth twice through in vitro fertilization and embryo transfer after endometrial carcinoma fertility preservation treatment. Int J Womens Health 6(16):395–400 10.2147/IJWH.S441984 PMC10924884 38463685

[24] Schilder JM, Thompson AM, DePriest PD et al (2002) Outcome of reproductive age women with stage IA or IC invasive epithelial ovarian cancer treated with fertility-sparing therapy. Gynecol Oncol 87(1):1–7 12468335 10.1006/gyno.2002.6805

